# Massive Open Online Course Versus Flipped Instruction: Impacts on Foreign Language Speaking Anxiety, Foreign Language Learning Motivation, and Learning Attitude

**DOI:** 10.3389/fpsyg.2022.833616

**Published:** 2022-02-07

**Authors:** Hui Pan, Fang Xia, Tribhuwan Kumar, Xiang Li, Atefeh Shamsy

**Affiliations:** ^1^School of Foreign Languages Studies, Wenzhou Medical University, Wenzhou, China; ^2^Xindeng High School, Hangzhou, China; ^3^Department of English Language and Literature, College of Science and Humanities at Sulail, Prince Sattam Bin Abdulaziz University, Al-Kharj, Saudi Arabia; ^4^School of Foreign Languages, Nanjing Agricultural University, Nanjing, China; ^5^Department of English, Islamic Azad University, Abadan, Iran

**Keywords:** MOOC, flipped instruction, speaking anxiety, foreign language learning motivation, attitude

## Abstract

This study inspected the effect of Massive Open Online Course (MOOC) and flipped instruction on EFL learners’ foreign language speaking anxiety, foreign language learning motivation, and attitude toward English learning. To fulfill this objective, the Oxford Quick Placement Test was given to 160 Iranian EFL learners, of whom 120 upper-intermediate participants were chosen and divided into two experimental groups—MOOC (*n* = 40) and flipped (*n* = 40)—and one control group (*n* = 40). After that, all selected participants were administered a speaking anxiety questionnaire and a motivation questionnaire as the pre-test of the research. Then, one of the experimental groups received an online-based instruction *via* Skype: one conversation was instructed to this group online every session. The other experimental group received the treatment *via* flipped-based instruction. The audio files and the texts of the conversations were sent to this group *via* the WhatsApp application because they all had easy access to it. On the other hand, the control group did not receive any Internet-delivered treatment yet was trained through a face-to-face method. This process continued until the last session, and after the treatment period, the post-tests of speaking anxiety and motivation were given to all three groups to determine the effectiveness of the treatment. Moreover, two attitude questionnaires were administered to the experimental groups to examine their attitudes toward the MOOC and the flipped models of instructions. The findings of the One-way ANOVA test, *Post-hoc* Scheffe test, and paired samples t-test showed that there were significant differences between the post-test of the experimental groups and the control group. The results indicated that the experimental groups significantly outflanked the control group after the treatment. Lastly, the outcomes showed that participants in both experimental groups had positive attitudes toward technological-based instructional environments.

## Introduction

Language learning is getting easier than before thanks to the miracle of the century—technology. Nowadays, different kinds of technology tools are seen everywhere, and they are frequently used in English language learning. One branch of technology is Massive Open Online Course (MOOC) that refers to online courses with free and open registrations, a publicly shared curriculum, and open-ended consequences. McAuley et al. (2010) state that MOOC integrates social networking and available online resources to help instructors in their teaching. In addition, MOOC builds on the involvement of students who self-organize their participation based on learning purposes, prior knowledge and skills, and common interests (McAuley et al., 2010).

Using MOOCs as an instructional instrument can provide a rich and varied learning context characterized by learners’ interactions from diverse locations. Its participatory, open, and innovative techniques supply new methods for the learners to learn in virtual learning environments (Navío-Marco and Solórzano-García, 2019). MOOC is a learning network empowered by the interactions among learners learning online that makes use of the new abilities and peculiarities of digital learning contexts (Navío-Marco and Solórzano-García, 2019).

The other branch of technology that can contribute to English learning is flipped instruction. Flipped instruction is an alternative instructional method that puts emphasis on student-centered instruction, keeping the conventional classroom environments as a reserve. It also highly captured interests and is welcome in high educational levels (Khayat et al., 2021). The flipped instruction is an incipient learning model that tries to boost learners’ active learning, collaboration, and support during the learning process *via* a better allocation of teaching time (Bergmann and Sams, 2012). Flipped instruction has lately captured much attention in both research and teaching. It is an educational approach that permits the instructors to make a video lecture, screencast describing the important concepts of the topic to learners and leaving the class time for more activity involvement under the guidance of the teachers (Milman, 2012). As a kind of student-centered learning approach, flipped instruction encompasses some theories and methods of constructivism and active learning with educational peers’ assistance (Chien-Yuan and Cheng-Huan, 2018).

The mentioned instructions, including MOOC and flipped, can be applied in EFL contexts to help learners increase their foreign language learning motivation. Motivation plays a vital role in anything, especially learning a foreign language. Motivation is differently defined based on different viewpoints. In language learning, an individual’s motivation is one of the agents which affect his/her success in learning the L2 (Tuan, 2012). This has been advocated by Sara et al. (2017), who argued that the primary agent influencing an L2 learners’ success is motivation. Motivation is vital to successful learning as it is an internal drive that will motivate students to perform activities to reach their objectives (Reyes, 2019). Similarly, Ryan and Deci (2002) hold that having motivation implies being moved to conduct a task or an activity. Contrary to unmotivated students who do not have the energy and inspiration to perform something, motivated students are energetic and dynamic to finish the tasks. Interest, curiosity, and desire to do something are the primary agents that compose motivated people (Williams and Burden, 1997).

The other psychological issue that the MOOC and the flipped instructions can be affected is speaking anxiety. Pertaub et al. (2001) state that anxiety generally comes out when the speakers are forced to give a public speech or communicate with foreign people because they have a fear of being evaluated or humiliated by others. Though people are cognizant that this nervousness is not rational, they cannot help feeling the anxiety that can lead to depressive disorder, distress, and irritation (Pertaub et al., 2001). Horwitz et al. (1986) hold that such anxiety simply inserts in the foreign language speaking process and may multiply when we communicate with native speakers of that language.

The MOOC and the flipped instructions can help EFL learners to form positive attitudes toward English language learning. Ellis (1994) asserts that having positive attitudes toward the second language and its users can be anticipated to develop learning, while having a negative attitude can hinder it. Learners’ attitudes have an effect on the foreign language proficiency levels of the EFL students. Thus, students with favorable attitudes get more successful in reaching their objective, while students with negative attitudes understand that acquiring a high level of English proficiency is difficult. In addition, even the students’ negative attitudes can be changed by using promising approaches and materials to assist pupils in gaining favorable attitudes toward the target language and the culture of that language (Brown, 2000). Doughty and Long (2003) hold that the acquisition of the L2 relies on a modification of the attitudes, knowledge, and behaviors toward the people of the target language. Positive changes in learners’ attitudes toward native speakers are vital for learning the target language. Students’ previous experiences as language learners can affect their attitudes. If they were successful, then they may be predisposed to success now. Failures then may imply that they expect failures now (Ahmed et al., 2015).

The psychological factors (motivation, anxiety, and attitude) explained above can be influenced by the online instructional methods of MOOC and flipped. Based on Huang and Hwang (2013), online materials can give impetus and motivation to the passive learners to learn the lessons actively *via* online instruction and therefore pay more attention to language learning. Online teaching puts emphasis on interactions between a teacher and students, unlike the traditional teaching methods. In order to increase students’ motivation and decrease their anxiety, different online tools and applications can be used in EFL contexts. Therefore, this study examined the effects of two types of online instructions, including MOOC and flipped, on Iranian EFL learners’ speaking anxiety and foreign language learning motivation.

## Materials and Methods

### Participants

To perform this investigation, the Oxford Quick Placement Test (OQPT) was administered to 160 Iranian EFL students, and 120 of them were chosen for the target population of the research. The subjects were chosen from three English Language Institutions of Esfahan city, Iran, at the upper-intermediate level. They were all male students because only males were accessible to the researchers. The chosen respondents were randomly divided into two experimental groups and a control group. One of the experimental groups was taught based on the MOOC instruction, and the other experimental group was instructed *via* flipped-based instruction, but the control group was trained *via* conventional instruction.

### Data Collection Instrumentations

The first tool applied in the current research was the OQPT which aided the researchers in choosing the homogenous students. This test had 100 items designed by the Oxford University Press and the University of Cambridge Local Examinations Syndicate. The test was validated in 21 countries by more than 7,000 learners, and its reliability reached 0.91 (Geranpayeh, 2003). This tool assesses reading comprehension (in the cloze test), vocabulary, and grammar knowledge. The scoring criteria for proficiency levels, according to Allan (2004), are as follows (Table 1).

**Table 1:** Scoring criteria of proficiency levels.

Proficiency levels	Cut-off points
Beginner	0–29
Breakthrough	30–39
Elementary	30–39
Lower-intermediate	50–59
Upper-intermediate	60–69
Advanced	70–79
Very advanced	80–100

The second instrument utilized in this research was the Speaking Anxiety Scale (SAS) that was an 18-item questionnaire extracted from Öztürk and Gürbüz (2014), who developed their questionnaire by choosing 18 items from the 33 items of the Foreign Language Classroom Anxiety Scale (FLCAS) designed by Horwitz et al. (1986). Öztürk and Gürbüz (2014) selected the 18 items among 33 FLCAS Horwitz et al.’s (1986) scale directly relevant to foreign language speaking anxiety. This questionnaire had 18 items, and each had five options: from Strongly Disagree to Strongly Agree. A panel of English professors confirmed the validity of this questionnaire, and its reliability was measured *via* utilizing Cronbach Alpha (*r* = 0.88).

The third instrument employed in this study was a questionnaire extracted from Gardner’s (2004) international version of “Motivation Test Battery” (MTB) to measure the students’ motivation. The original test battery has 12 scales with 104 items merged into six variables. However, in the present research, the questionnaire items focused on assessing integrative motivation, instrumental motivation, attitudes toward learning situations, and learners’ motivation with 74 items. The scale applied in the questionnaire was a five-point Likert-type scale from strongly disagree to strongly agree. Some English instructors verified the FLCAS’s validity, and its reliability was measured by applying Cronbach Alpha (*r* = 0.85). It should be noted that the SAS and the MTB questionnaires were utilized twice in this research: once as the pre-test and once as the post-test.

The other tool employed in the current investigation was an attitude questionnaire admonished to the MOOC group to examine their ideas about MOOC-based instruction. This tool was made by the researchers and had 20-point Likert-type items. The researchers computed the reliability of this tool by using Cronbach’s alpha (*r* = 0.86).

The fifth tool employed in the current investigation was a questionnaire given to the flipped participants to examine their opinions toward utilizing flipped classrooms. The questionnaire of Hashemifardnia et al. (2021) containing 20 Likert scale statements was used for this purpose. The reliability of this questionnaire was measured through Cronbach’s alpha (*r* = 0.83). It is worth mentioning that some English professors confirmed the validity of both attitude questionnaires.

### Procedures

To perform this research, 120 homogenous participants were chosen and divided into three groups of 40: two experimental (MOOC and flipped) and a control. Then, the questionnaires of MTB and SAS were administered to examine the motivation and speaking anxiety of the respondents before starting the instruction. After that, one experimental group received the treatment by using an online-based instruction-MOOC. In every session, one conversation was instructed *via* the MOOC program to the participants of this group. In the MOOC-based class, both teachers and students simultaneously worked in a learning context. The researchers set an exact time for an online class, and participating for all participants was an obligation. The students chatted and discussed the materials in an online setting. Everything was done on the online platform. It must be stated that after the instruction, a questionnaire was administered to the MOOC respondents to examine their opinions about MOOC instruction implementation.

The flipped group was sent the materials (conversations) *via* the WhatsApp application, and they were accountable for their own learning. The participants of this group were asked to study the materials before coming to the real class. Three or four days before each face-to-face class, the researcher sent the audio files and the texts of the conversations to students *via* the WhatsApp application. The learners were asked to read and practice the conversations prior to attending the classes. After attending the class, the researcher extracted some information pertinent to the conversations from students. Moreover, some students were randomly chosen to conduct the conversation in front of the class. A questionnaire was given to examine the flipped participants’ ideas about applying the flipped instruction when all conversations were taught.

The control group participants were traditionally instructed the conversations without using any online instruction. First, the researcher provided the students with some information about the topic of the conversation, and then, he played the audio file two or three times; after listening to the audio file and teaching each conversation, the participants were wanted to practice it with their classmates and carry it out in front of the students. After teaching 10 conversations, the questionnaires of MTB and SAS were readministered to investigate the influences of the instruction on the pupils’ motivation and speaking anxiety.

### Data Analyses

After gathering the data *via* the pre-tests, the post-tests, and the questionnaires, they were analyzed using the SPSS software, version 22. Firstly, the One-way ANOVA test and *Post-hoc* Scheffe test were employed to analyze the performances of the three groups before and after the treatment. Secondly, a One-sample t-test was applied for analyzing the data collected *via* the attitude questionnaires.

## Review of the Literature

### Theoretical Background

Nowadays, the growth of technology and the incorporation of new technological attainments are observed in our daily lives (Kalogiannakis and Papadakis, 2019). Technology and computer are used daily to help us perform our tasks more efficiently. Technology has affected all aspects of our lives, particularly our instructional contexts and how the instructional process occurs; it also has made some changes in traditional teaching classes (Papadakis et al., 2020). Technologies are considered the agent of improving the process of teaching and learning (Papadakis et al., 2016).

One technological-based teaching is flipped instruction which has gotten significantly common in educational contexts, and they more universally utilized by instructors in the past several decades (Moranski and Kim, 2016); it is urgently needed to study the impacts of flipped instruction on the language learning process. The flipped instruction makes the conventional classroom reversed by showing the educational materials online before the class and then makes students involved in interactive group learning or critical problem-solving activities conducted with the help of instructors in the classroom (Herreid and Schiller, 2013).

The flipped classroom can be considered as a kind of student-centered learning since students are responsible for learning independently by flipped materials facilitated by instructors and boosting more participation in discussions and research activities in the classrooms (Williams et al., 2019). In this way, the learners obtain greater roles and responsibilities in their own learning processes. This structure allows the classroom to clarify contents by some explanations (Touron and Santiago, 2015).

The other technological-based teaching is MOOC which is a desirable mode of instruction. As its name shows, MOOC is a method in which instructional content is presented online to those students who have online courses, without limitations on attendance (MacLeod et al., 2015). MOOC is referred to the online courses presented utilizing various media, such as video, forums, and resources, to countless students willing to study in pick universities (Baturay, 2015). “MOOC as a novel form of online learning was applied to explain online open courses that were improved at the University of Manitoba by George Siemens and Stephen Downes” (Mellati and Khademi, 2018, p. 3). Connectivism was the first online course introduced by Siemens and Downes in 2008. They held that “networks shape knowledge, and learning is a process for connecting specific nodes and information resources” (Li, 2017, p. 11). Connectivism is a theory that supports MOOC; according to it, connections make the exchange of knowledge simpler, and all pupils can contribute to knowledge imparting (Waks, 2016). Connectivists believed that knowledge is not just conveyed from the teacher to the student and learning does not happen in a single context; instead, they stated that knowledge is transmitted by individual interaction, principally in the web environments (Kop, 2011). Connectivists believe that learners are accountable for their learning. Following the Connectivists ideas, in MOOC, learners make and manage their learning (Kesima and Altınpulluka, 2015).

Massive open online course and flipped instructions can help EFL learners to raise their foreign language learning motivation. EFL learners’ motivational orientation is the psychological condition that shows their needs, desires, and aims to learn a target language expressed *via* specific actions. Based on Brown (2000), motivation is likely the most often applied catch-all term for describing the successes or failures of virtually all complicated tasks. Ellis (1997) believed that motivation is naturally dynamic; it is not something that learners have or do not have but rather something different from one moment to the next depending on the learning situations or tasks.

Muftah and Rafic-Galea (2013) stated that motivated learners would be more enthusiastic and willing to dedicate time to language learning. Thus, barriers in learning a language may be caused by a lack of motivation and a negative attitude (Oroujlou and Vahedi, 2011). Nasri et al. (2021) stated that students are motivated to learn a foreign language based on three reasons, including internal causes (i.e., an individual’s attention in a foreign language); integrative causes (i.e., involvement in other cultures or communicating with other individuals); and instrumental causes (i.e., a person’s self-benefit like career advancement).

In addition to motivation, EFL learners’ foreign language speaking anxiety can be influenced by new teaching methods, such as MOOC and flipped instructions. Anxiety can facilitate and impede the language learning process (Aydin, 2018). To date, research findings generally show that anxiety negatively impacts the foreign language learning process (Ellis, 2012). MacIntyre (1995) explained that nervous learners are focused on both tasks at hand and their reactions to them; they will not learn as rapidly as relaxed learners. This is in accordance with Krashen’s affective filter hypothesis (1985), which proposed that learning could only occur when a learner’s affective filter is not blocking the process. Less motivated students with high anxiety levels need higher affective filters that impede input and learning (Ellis, 2012).

Having less anxiety during learning can assist EFL learners in having positive attitudes toward English language learning. Attitude has lately received much attention from both first and second language scholars. The majority of the studies on the issue concluded that students’ attitudes are a crucial part of learning and must become an integral part of second or foreign language learning pedagogy. There are some reasons why investigating students’ attitudes toward language learning is significant. First, attitudes toward learning are supposed to affect behavior (Weinburgh, 1998). Second, a correlation between attitudes and successes or achievements has been indicated to exist. Schibeci and Riley (1986), as cited in Weinburgh (1998), reported support for the hypothesis that attitude influences achievements rather than achievements affecting attitude. The reason is that attitude affects one’s behaviors, inner moods, and learning. Thus, it is evident that there is a relation between language learning and the environmental elements in which the students grow up.

### Empirical Background

In this part, some experimental studies on the effects of the MOOC and the flipped instructions on developing language learning are reported. For example, Ventura and Martín-Monje (2016) examined the impact of Facebook in a MOOC environment on learning technical vocabularies. A mixed-method approach was utilized for collecting the needed data. The results indicated that using the MOOC environment positively affected the students’ motivation to learn technical vocabulary.

Padilla Rodriguez and Armellini (2017) carried out research on enhancing students’ self-efficacy *via* applying MOOC instruction. To reach this aim, 32 subjects from two countries of Mexico and Colombia were chosen. At the beginning and the end of the treatment, the subjects were required to answer a questionnaire encompassing the General Self-Efficacy Scale, items on specific study skills, and space for non-mandatory comments. The results displayed a noticeable improvement in general self-efficacy after the treatment and the perceived self-efficacy pertinent to five out of six study skills. The outcomes also indicated that MOOCs could represent low-risk, formative opportunities to enhance the respondents’ knowledge and improve self-efficacy.

Hashemifardnia et al. (2020) investigated the impacts of MOOCs on developing Iranian EFL students’ speaking complexity, accuracy, and fluency (CAF). First, they selected 60 intermediate participants; second, they divided them into experimental and control groups. After that, the researchers gave a speaking pre-test to both groups. After that, the experimental participants were trained in some conversations by an online-based instruction through Skype. On the other hand, the control respondents were traditionally taught in the conversations without using online instruction. After the instruction, an attitude questionnaire was given to the MOOC subjects to examine their general opinions about utilizing the MOOC instruction. The outcomes confirmed the MOOC participants remarkably outflanked the control participants on the speaking post-test. Furthermore, the one-sample t-test indicated that the participants had desirable attitudes toward implementing the MOOC instruction in English learning.

Recently, Sudarmaji et al. (2021) scrutinized the flipped instruction’s impact on learners’ speaking skills. To do this research, 34 senior high school students were chosen as the subjects of the investigation. The subjects were assigned to two groups: flipped and non-flipped. A pre-test of speaking was given prior to the treatment, and a speaking post-test was used after the flipped instruction. The results showed that the online flipped classroom model significantly improved the students’ speaking performances after the treatment.

Hashemifardnia et al. (2021) studied the effect of utilizing flipped classrooms on helping EFL students develop their speaking CAF. To achieve this purpose, 60 intermediate EFL learners were chosen and assigned to flipped and non-flipped. Then, all participants were administered a speaking pre-test. Later, the flipped participants received the treatment *via* flipped-based instruction, but the traditionally trained non-flipped participants. At the end of the treatment, an attitude questionnaire was given to the flipped participants to check their opinions about applying to the flipped classroom. The outcomes proved that the flipped group did better than the non-flipped group on the speaking post-test. In addition, the findings revealed that the participants presented desirable attitudes toward using flipped instruction in English language learning.

After reviewing the related literature, it was revealed that most investigations inspected the influences of the MOOC and the flipped instructions on vocabulary, reading skills, and speaking skills. There are few experimental investigations on the MOOC’s effect and flipped instructions on Iranian EFL learners’ speaking anxiety and foreign language learning motivation. Thus, the below questions were formulated to cover this gap.

**RQ1.** Does MOOC instruction bear any significant impact on Iranian EFL learners’ speaking anxiety and foreign language learning motivation?
**RQ2.** Does flipped instruction bear any significant impact on Iranian EFL learners’ speaking anxiety and foreign language learning motivation?
**RQ3.** Do Iranian EFL learners have positive attitudes toward using the MOOC instruction in learning the English language?
**RQ4.** Do Iranian EFL learners have positive attitudes toward using flipped instruction to learn English?

## Results

In this section, the collected data were analyzed, and the gained results are presented in the following tables:

### Effects of the MOOC and Flipped Instructions on EFL Learners’ Speaking Anxiety

The data gathered *via* the anxiety questionnaire administering before and after the treatment were analyzed in this part.

Based on Table 2, the control group’s mean score is 44.52, the flipped group’s mean score is 45.12, and the MOOC group’s mean score is 43.87. This table shows that Sig. (0.94) is higher than (0.05); therefore, the differences between the groups are not noticeable at (*p* < 0.05). Indeed, the three groups had the same level of speaking anxiety before the treatment.

**Table 2:** ANOVA results for anxiety pre-test.

Groups	*N*	Means	*SD*	Std. errors	
Control	40	44.52	16.49	2.60	
Flipped	40	45.12	17.44	2.75	
MOOC	40	43.87	16.18	2.55	
Total	120	44.50	16.58	1.51	
Source of variation	Sum of squares	***df***	Mean square	***F***	***p***
Between group	31.26	2	15.63	0.05	0.94
Within groups	32702.72	117	279.51		
Total	32733.99	119ss			

The above table indicates that the mean score of the control participants is 45.37, the mean of the flipped participants is 72.27, and the mean score of the MOOC group is 70.95. Based on Table 3, the difference between the anxiety post-tests of the participants is remarkable because Sig. (0.00) is smaller than (0.05); thus, it can be said that the experimental participants outflanked the control participants in the post-test of anxiety.

**Table 3:** ANOVA results for anxiety post-test.

Groups	*N*	Means	*SD*	Std. errors	
Control	40	45.37	17.71	2.80	
Flipped	40	72.27	9.84	1.55	
MOOC	40	70.95	10.13	1.60	
Total	120	62.86	17.96	1.64	
Source of variation	Sum of squares	***df***	Mean square	***F***	***p***
Between group	18392.61	2	9196.30	53.72	0.00
Within groups	20029.25	117	171.19		
Total	38421.86	119			

Table 4 compares the performances of all groups in the anxiety post-tests. This table reveals noticeable differences between the post-test of the control participants and the post-tests of both experimental participants (*p* < 0.05). Moreover, the outcomes show that there were no significant differences between the scores of both experimental groups in the anxiety post-tests.

**Table 4:** *Post hoc* Scheffe test, multiple comparisons (anxiety post-tests).

(I) groups	(J) groups	Mean differences (I-J)	Std. errors	*p*
Control	Flipped	−26.90	2.92	0.00
	MOOC	−25.57	2.92	0.00
Flipped	Control	26.90	2.92	0.00
	MOOC	1.32	2.92	0.90
MOOC	Control	25.57	2.92	0.00
	Flipped	−1.32	2.92	0.90

### Effects of the MOOC and Flipped Instructions on EFL Learners’ Motivation

The following Table 5**–**Table 7 show the results related to the effects of the MOOC and flipped instructions on Iranian EFL learners’ foreign language motivation learning.

**Table 5:** ANOVA results for motivation pre-test.

Groups	*N*	Means	*SD*	Std. errors	
Control	40	114.10	33.72	5.33	
Flipped	40	117.27	34.54	5.46	
MOOC	40	115.72	37.29	5.89	
Total	120	115.70	34.94	3.19	
Source of variation	Sum of squares	***df***	Mean square	***F***	***p***
Between group	201.650	2	100.825	0.08	0.92
Within groups	145151.550	117	1240.612		
Total	145353.200	119			

**Table 6:** ANOVA results for motivation post-test.

Groups	*N*	Means	*SD*	Std. errors	
Control	40	117.32	37.43	5.91	
Flipped	40	297.87	30.98	4.89	
MOOC	40	295.88	30.03	4.74	
Total	120	237.02	91.07	8.31	
Source of variation	Sum of squares	***df***	Mean square	***F***	***p***
Between group	859765.40	2	429882.70	395.20	0.00
Within groups	127265.52	117	1087.74		
Total	987030.92	119			

**Table 7:** *Post-hoc* Scheffe test, multiple comparisons (motivation post-tests).

(I) groups	(J) groups	Mean difference (I-J)	Std. error	*p*
Con	Flipped	−180.55	7.37	0.00
	MOOC	−178.55	7.37	0.00
Flipped	Control	180.55	7.37	0.00
	MOOC	2.00	7.37	0.96
MOOC	Control	178.55	7.37	0.00
	Flipped	−2.00	7.37	0.96

Table 5 displays the mean scores of all three groups in the motivation pre-test. The control group’s mean scores, the flipped group, and the MOOC group are 114.10, 117. 27, and 115.72, respectively. According to the results presented in the above table, the difference between the motivation pre-tests of the three groups is not remarkable because Sig. (0.92) is greater than 0.05.

Table 6 shows that the control group’s mean scores, the flipped group, and the MOOC group are 117.32, 297.87, and 295.88, respectively. It appears that experimental participants outflanked the control participants in the post-test of motivation. According to the findings in the above table, the difference between the motivation post-tests of the three groups is noticeable as Sig. (0.00) is smaller than (0.05); thus, we can say that the experimental groups had better performances than the control group in the motivation post-test.

In Table 7, the performances of the three groups in the motivation post-tests are compared. The results reveal that there were significant differences between the motivation post-test of the control group and the post-tests of both experimental groups (*p* < 0.05); besides, the results indicate that there was not a remarkable difference between the motivation post-tests of both experimental participants.

### Attitudes of EFL Learners Toward the MOOC Instruction

In this section, the data collected *via* the MOOC questionnaire were analyzed to check Iranian EFL learners’ attitudes toward the MOOC instruction.

As revealed in Table 8, the significance level is 0.00 (Sig. = 0.00), smaller than 0.05. This implies that the respondents of this investigation held favorable attitudes about the MOOC instruction.

**Table 8:** One-sample test of the MOOC questionnaire.

Test value = 0					
*t*	*df*	Sig. (two-tailed)	Mean differences	95% confidence interval of the differences	
				Lower	Upper
58.69	19	0.00	3.50	3.18	3.52

### Attitudes of EFL Learners Toward the Flipped Instruction

In this part, we analyzed the data gathered through administering the flipped questionnaire after the treatment to examine the participants’ attitudes toward flipped instruction.

As displayed in Table 9, the significance level is 0.00 (Sig. = 0.000) smaller than 0.05, indicating that the respondents in the current investigation presented desirable attitudes toward using the flipped instruction.

**Table 9:** One-sample test of the flipped questionnaire.

Test value = 0					
*t*	*df*	Sig. (two-tailed)	Mean differences	95% confidence interval of the differences	
				Lower	Upper
49.91	19	0.00	3.49	3.86	3.83

## Discussion of the Study

Regarding the first question of the study, the results showed that the experimental participants who had received MOOC instruction outflanked the control participants who had been deprived of the MOOC instruction. This improvement and betterment can be attributed to the MOOC instruction since it can save time and effort. MOOC instruction was open to students and gave them more opportunities to express themselves.

By teaching *via* MOOC, more rooms can be provided for the students to share their ideas and knowledge. Also, MOOC-based instruction can supply comfortable contexts for learners to establish interactions and communications with their classmates and instructors. In addition, in a MOOC-based setting, learners can access the material and feedback even long after the course (Richter and McPherson, 2012). Furthermore, a MOOC-based setting can aid the learners to learn the English language more independently. The benefits mentioned for the MOOC-based instructional setting can be why the MOOC participants gained better scores in their post-test compared to the control participants. In other words, the advantages reported for the MOOC-based instruction can be the reasons for the findings obtained in this study.

The findings of this study confirm the study of Hashemifardnia et al. (2020), who examined the effects of the MOOC instruction on Iranian EFL students’ speaking CAF and indicated that using the MOOC instruction had a significant effect on developing the participants’ CAF. This investigation is advocated by the results of Ventura and Martín-Monje (2016), who verified the positive influences of the MOOC instruction on the students’ motivation to boost their technical vocabulary knowledge. Moreover, Padilla Rodriguez and Armellini (2017) supported this research, who found that their respondents’ self-efficacy improved remarkably after the MOOC treatment. This research supports the Connectivism theory saying that sharing knowledge, social network, and open educational resources can make language learning simpler (Siemens, 2005).

The other reasons for the obtaining results in this study can be that the MOOC-based instruction allows the students to have communications with other learners whenever they like, permits them to learn English at any time and area, provides them the opportunities to access many materials even after the classes, helps them manage their learning, develops their independence, and decreases their anxiety levels (Tatiana Dina and Ciornei, 2013). In addition, the MOOC-based instruction can recommend that the students participate actively in tasks beyond the classrooms and coursebooks, send messages to the native speakers, and communicate in the target language.

Concerning the second research question, the outcomes demonstrated that the flipped participants did better than the control participants in the anxiety post-test. We can ascribe this improvement to the advantages of flipped instruction. The amalgamation of face-to-face instruction and flipped instruction can prepare a suitable situation for learners to learn the English language more successfully. The obtained outcomes in this research show that technological improvements can shift the processes of English learning and teaching into a better way, as Wells et al. (2008) claimed that technological-based methods have remarkably changed the ways teachers instruct and students learn. Also, flipped instruction can foster cooperative learning among the learners as it is a student-centered model; consequently, pupils have more opportunities to work cooperatively to learn novel materials better. Moreover, the flipped setting involves learners in one-on-one activities that develop critical thinking skills and promote students’ communication (Farrah and Qawasmeh, 2018).

One reason for the gained outcomes can be that in the flipped instruction, the responsibility of learning is on the shoulders of the students as Harris et al. (2016) asserted that the flipped method puts the learning responsibility upon the learners’ shoulders; teachers become the specialists who enhance the talents of their students and remove the codependency. Flipped instruction is a student-centered situation that replaces the conventional lecture by activating collaborative tasks applying Internet and computer technology to transfer the materials out of the classroom in a way that permits students to learn cooperatively with their peers before coming to the face-to-face class.

The outcomes of the current study lend support to the findings of Sudarmaji et al. (2021), who examined the impacts of applying the flipped classroom on students’ speaking skills. Their outcomes showed that the online flipped model significantly developed the learners’ speaking performances. In addition, our study findings are in line with Hashemifardnia et al. (2021), who inspected the effect of the flipped instruction on Iranian EFL students’ speaking CAF and confirmed that there were remarkable differences between the post-test of the experimental and the control participants in favor of the experimental participants.

Furthermore, the current study’s findings are congruent with Rajabi et al. (2021), who inspected the effect of flipped instruction on Iranian EFL students’ classroom anxiety and listening performance. They indicated that the experimental participants obtained better scores on the listening pre-test, meaning that the flipped instruction model developed Iranian EFL learners’ listening comprehension. This study is also advocated by Hsieh et al. (2016), who examined the impacts of the flipped classroom on learners’ idiomatic knowledge and their oral ability. Their results indicated that flipped classrooms helped the learners improve their idiomatic knowledge and oral ability.

Concerning the third research question, the outcomes of the one-sample test indicated that Iranian EFL students had positive attitudes toward applying the MOOC instruction. MOOC instruction can aid learners in learning English both inside and outside of the class. Learners and many individuals across the globe utilize the MOOCs to learn for different reasons, such as: “job development, changing job, college preparation, supplemental learning, lifelong learning, and corporative learning and training (Devi, 2020, p. 1).” The reported features of MOOC can be the reasons why the students displayed positive attitudes toward implementing the MOOC instruction.

This research is congruent with Joseph and Nath (2013), who inspected Indian learners’ attitudes toward MOOC instruction. They figured out that 66% of the subjects strongly suggested that the MOOC courses should be used in their university. Also, this investigation is supported by Alanazi and Walker-Gleaves (2019), who discovered that learners held positive attitudes toward utilizing the Hybrid MOOCs with Flipped Classrooms. In addition, the findings of this investigation advocate the outcomes of Sahli and Bouhass Benaissi (2018), who showed that the respondents in their study presented positive attitudes toward online instructions in teaching writing skills. Also, the findings of this research are advocated by Hashemifardnia et al. (2020), whose results displayed that Iranian EFL students had significantly desirable attitudes toward using MOOC instruction for speaking classes.

The positive attitudes of Iranian learners toward applying the MOOC instruction can be due to the possibility that they feel more comfortable expressing themselves online. Moreover, the accessibility of the MOOC instruction and easy access to online courses helped EFL learners to form positive attitudes toward the MOOC instruction. The other probable reason for presenting positive attitudes toward MOOC-based instruction can refer to the learners’ more contact with their teachers and classmates at any time (Hashemifardnia et al., 2021).

Respecting the fourth research question, “What are Iranian EFL learners’ attitudes toward using the flipped instruction in learning the English language?” the outcomes of the one-sample test indicated that Iranian EFL students showed positive opinions about implementing the flipped instruction. After teaching and learning *via* flipped instruction, the learners showed an inclination toward it. The reason why the learners showed positive attitudes toward using the flipped instruction might refer to the interactions, cooperation, and independency the flipped instruction supplied as AlJaser (2017) believed applying flipped classroom can provide learners with more opportunities for interactions and responsibilities toward learning. Also, Sirakaya and Ozdemir (2018) asserted that pupils welcome the employment of flipped instruction because it boosted their motivation, cooperation, and achievement.

The results of this research are endorsed by Farrah and Qawasmeh (2018) who investigated EFL students’ attitudes toward applying the flipped classroom. Their results showed that EFL students had positive attitudes toward using the flipped classroom. Similarly, this research lends support to the results of Marlowe (2012), who figured out that learners in her investigation had positive attitudes toward using the flipped classroom.

We can attribute the obtained outcomes to the flipped classroom model’s characteristics that follow the procedures that make learners accountable for their learning independently before coming to the real class sessions *via* practicing materials and discussing in the social network groups. In addition, using the flipped classroom model can free the educational time and facilitate the path for interactive learning activities that improve the learners’ communicative competencies.

## Conclusion and Implications of the Study

This investigation inspected the MOOC’s impacts and the flipped instructions on Iranian EFL learners’ speaking anxiety and foreign language learning motivation. According to the findings, we can conclude that using the mentioned instructions could help Iranian EFL students develop their foreign language learning motivation and reduce their speaking anxiety. In addition, we conclude that Iranian EFL students presented favorable attitudes toward utilizing both MOOC and flipped instructions. Using online instructions besides face-to-face instructions can produce positive consequences for EFL students. This research concludes that the MOOC and the flipped models can provide more opportunities to learn English both inside and outside the class milieu while removing the restrictions on time and place, which are common in conventional classes. In addition, using flipped classrooms can facilitate class discussion, promote autonomy and self-direction of the learners (Zainuddin and Perera, 2018). Eventually, it can increase students’ academic achievements, enhance learners’ collaborative and communicative skills, and decrease students’ anxiety (Farrah and Qawasmeh, 2018). Regarding the advantages and benefits enumerated for a flipped classroom, it is seriously suggested that the Iranian educational system implements this teaching model in the students’ curriculum.

The results of this study can produce some pedagogical implications for instructors, learners, and material designers to take the benefits of the online instructions into account. Therefore, this research can encourage English teachers to implement technological-based methods in their classrooms in order to gain better educational achievements. Also, the flipped classroom can encourage instructors and teachers to recommend a many-sided and appealing method to exchange learning content while permitting students to monitor their own learning processes.

Students can benefit from the findings of this research; students who cannot attend the classes due to their problems (illness and long-distance) can use the MOOC and the flipped instructions to compensate for their absence. The MOOC and the flipped instructions make the students responsible for their own learning. By supplying lectures online, instructors can pave the way for the learners to learn the lessons at their own speed. This research can assist those learners who have embarrassment in participating in face-to-face classes. In addition, the outcomes of this research can persuade the material designers to integrate online instructions into EFL syllabuses.

## Limitations and Suggestions of the Study

Though we try to conduct this research ideally, drawbacks are unavoidable in any empirical investigation. Including a small sample (120 Iranian upper-intermediate EFL students) was the main limitation of this study; therefore, we should generalize the results to other populations carefully. Further studies can include more participants to get richer findings. Only quantitative instruments were used to collect the data for answering the research questions; upcoming research is proposed to apply both qualitative and quantitative instruments to boost the reliability and validity of the results. The subjects of this study were only males; future studies are recommended to include females, too. Future investigations can expand the treatment time and investigate the effect of the MOOC and the flipped instructions on different skills in different settings.

## Data Availability Statement

The datasets presented in this study can be found in online repositories. The names of the repository/repositories and accession number(s) can be found in the article/supplementary material.

## Author Contributions

All authors listed have made a substantial, direct, and intellectual contribution to the work, and approved it for publication.

## Conflict of Interest

The authors declare that the research was conducted in the absence of any commercial or financial relationships that could be construed as a potential conflict of interest.

## Publisher’s Note

All claims expressed in this article are solely those of the authors and do not necessarily represent those of their affiliated organizations, or those of the publisher, the editors and the reviewers. Any product that may be evaluated in this article, or claim that may be made by its manufacturer, is not guaranteed or endorsed by the publisher.
